# Assessment of the Knowledge, Attitude, and Perception (KAP) of Sheep Farmers Regarding Ticks and Tick-Borne Pathogens in Tunisia, North Africa

**DOI:** 10.3390/vetsci12010002

**Published:** 2024-12-26

**Authors:** Médiha Khamassi Khbou, Syrine Rekik, Rihab Romdhane, Limam Sassi, Felicitas Bergmann, Martin H. Groschup, Mourad Rekik, Mohamed Gharbi

**Affiliations:** 1 Laboratory of Infectious Animal Diseases, Zoonoses, and Sanitary Regulation, Institution of Agricultural Research and Higher Education, National School of Veterinary Medicine of Sidi Thabet, University of Manouba, Sidi Thabet 2020, Tunisia; 2 Laboratory of Parasitology, Zoonoses, and Sanitary Regulation, Institution of Agricultural Research and Higher Education, National School of Veterinary Medicine of Sidi Thabet, University of Manouba, Sidi Thabet 2020, Tunisia; rekiksyrine@yahoo.com (S.R.); rihabromdhaneveto@gmail.com (R.R.); sassilimam@yahoo.fr (L.S.); gharbim2000@yahoo.fr (M.G.); 3 Friedrich-Loeffler-Institut, Institute of Novel and Emerging Infectious Diseases, Südufer 10, 17493 Greifswald, Insel Riems, Germany; felicitas.bergmann@fli.de (F.B.); martin.groschup@fli.de (M.H.G.); 4 International Center for Agricultural Research in the Dry Areas (ICARDA), Avenue Hédi Karray, Ariana 2049, Tunisia; m.rekik@cgiar.org

**Keywords:** ectoparasites, North Africa, questionnaire

## Abstract

Ticks and tick-borne pathogens (TBPs) are a threat to human and animal health worldwide. A structured questionnaire was used to assess the knowledge, attitudes/practices, and perceptions (KAP) of 86 farmers of extensive sheep farming systems across different agro-ecological areas in Tunisia. The response rate was about 91.3%. Overall, 68.5% of the questions referring to knowledge and perceptions were answered correctly. Indeed, about half of the respondents were aware that ticks infest animals, with weight loss given as the major consequence. However, more than half of the farmers were unaware of the transmission and vectorial role of ticks. Those who knew that ticks are vectors cited microbes and icterus as the main concerns. A broad majority of farmers (70.9%) stated that they removed the attached ticks manually and 45.3% crushed them. As acaricides were perceived to be efficient for fighting ticks, according to 97.7% of the sheep farmers, they were used for both the animals and their sleeping areas. Although the toxicity of acaricides is known, 59.3% of the respondents did not use personal protection equipment when applying these products. Taken together, gaps in KAP among sheep farmers were identified. It can be used to better design awareness communication tools for TBPs.

## Simple Summary

A questionnaire on the knowledge, attitudes/practices, and perceptions was answered by 86 sheep farmers from Tunisia. The aim was to identify potential gaps in relation to ticks and tick-borne pathogens in order to better design communication tools to raise awareness among farmers. Overall, the majority of the questions on knowledge and perceptions were answered correctly. However, a high proportion of the farmers was not aware of the transmission and vector role of ticks. The questions on attitudes and practices were answered positively by half of the study participants. Especially questions on the removal of ticks (e.g., manually, using acaricides) and the handling of acaricides were quite difficult for the sheep farmers. The results can serve as a guideline for how to implement efficient control measurements.

## 1. Introduction

Tick-borne diseases are caused by different pathogens, including bacteria such as *Rickettsia*, *Ehrlichia*, and *Borrelia*, protozoa such as *Babesia* and *Theileria*, and several viruses. These pathogens can lead to serious illness or death in the affected animals, which in turn causes substantial losses in agricultural productivity [1]. The economic toll is not limited to the direct health costs but also extends to reduced fertility, weight loss, and lower milk production in the infected livestock [2,3,4]. Beyond their impact on animal health, tick-borne pathogens (TBPs) represent a significant threat to human populations. Many of these TBPs, such as *Borrelia burgdorferi* s.l., Crimean–Congo haemorrhagic fever virus, and tick-borne encephalitis virus, are zoonotic, meaning they can be transmitted from animals to humans [5]. Humans typically become accidental hosts when they are bitten by infected ticks in areas such as rural or forested regions [6]. In some cases, transmission can also occur through blood transfusions, although this is less common [1]. Furthermore, the incidence of tick-borne diseases has been increasing in recent years, driven by factors such as climate change, which is expanding the habitats suitable for tick populations, and shifts in land use, which increase human and livestock exposure to ticks [6]. To fight this growing threat, several strategies have been developed to control tick populations and limit the spread of tick-borne diseases. These methods include the use of chemical repellents and acaricides to kill or deter ticks, habitat management practices to reduce tick habitats, and personal protection measures to prevent tick bites [7]. In addition, researchers are exploring ways to breed livestock that are more naturally resistant to tick infestations. Moreover, efforts are being made to develop effective vaccines to protect both animals and humans from tick-borne pathogens [8]. However, despite the wide range of control measures available, managing tick populations and the diseases they carry remains a complex challenge. The development of resistance to acaricides, for instance, has made it harder to rely solely on chemical treatments, and the improper use of these chemicals often reduces their effectiveness [9]. These challenges highlight the urgent need for more sustainable, long-term solutions that integrate multiple approaches to tick management. A crucial aspect of improving tick control efforts lies in understanding the knowledge, attitudes, and practices (KAP) of farmers. Since farmers are the ones directly affected by tick infestations and are responsible for implementing control measures, their perspectives and behaviours play a critical role in the success or failure of tick management strategies. Gathering insights into what farmers know about ticks, how they perceive the risks associated with tick-borne diseases, and what actions they take to protect their livestock is essential for developing effective, evidence-based interventions. While numerous studies have been conducted on this topic globally, there is a lack of research focusing on sheep farmers in Tunisia, particularly across its diverse agro-ecological zones. This is an important gap, as the farming practices and the environmental conditions in these areas can have a significant impact on tick prevalence and the effectiveness of control measures [10]. Additionally, local cultural and economic factors may influence how the farmers perceive and respond to tick-related challenges. This study, carried out among extensively managed sheep farmers in Tunisia, aims to address this gap of knowledge by providing a comprehensive assessment of their KAP regarding ticks and the pathogens they transmit. The primary objective is to generate data that can be used to develop targeted strategies for improving tick prevention and control in Tunisia and similar regions.

## 2. Materials and Methods

### 2.1. Study Areas

**Figure 1:**
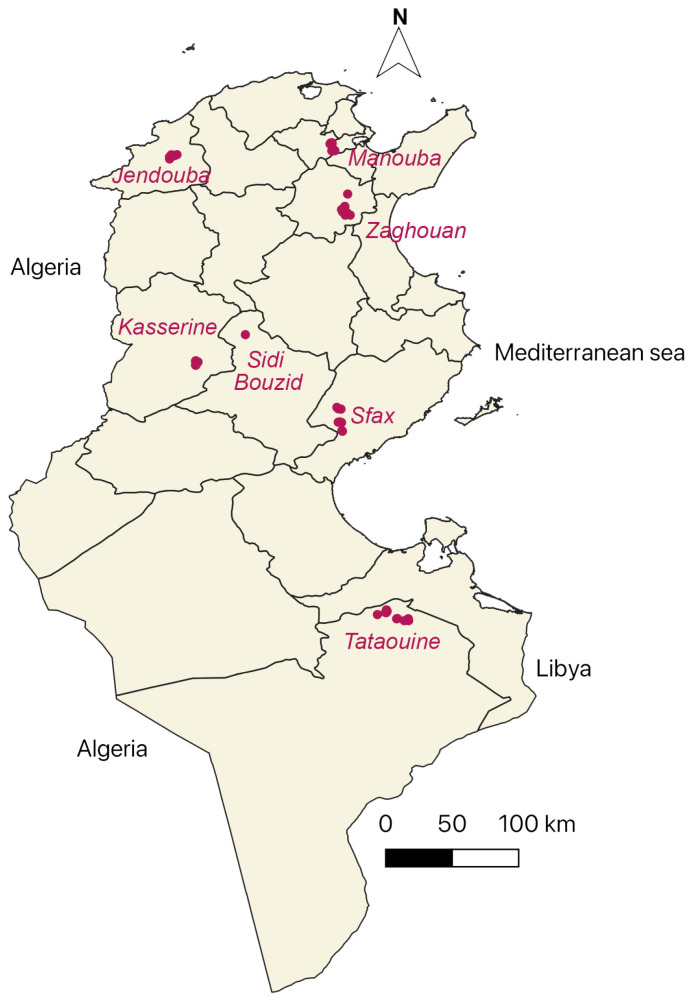
Map of Tunisia, showing in red dots the localities where sheep owners were interviewed [12].

The present KAP study was carried out on small ruminants’ owners in parallel with another work on ticks and tick-borne pathogens conducted between 2018 and 2020 in the six representative regions of the most predominant Tunisian agro-ecological areas (Figure 1) [11]. Farmers included in this study were selected based on their geographic distribution. Farmers were selected based on where their animals were grazing at the time of the survey. All chosen farmers represented the extensive farming system of small ruminants, although it should be noted that the climate in Tunisia varies between the North (Mediterranean climate zone: humid to sub-humid) the centre (central steppe climate zone: semi-arid to arid) and the South (southern desert climate zone: desertic).

### 2.2. Questionnaire and Data Collection

A structured questionnaire was designed in order to collect data on herd management and to assess the knowledge, the attitudes, and the practices of sheep farmers in Tunisia regarding ticks and tick-borne pathogens. The questionnaire initially comprised 60 questions: 23 (38.3%) were multiple choice, 15 (25%) were “yes or no”, 6 (10%) were one choice, and 8 (13.3%) were open questions. Eight questions were observed and registered without asking farmers (time of starting and ending questionnaire, the date, the governorate’s name, the village’s name, and GPS coordinates). The multiple-choice questions were not given as possible answers to the farmers and the option “Other” was always possible for all questions. When removing the questions related to “Other”, 40 questions were left and analysed for the present paper. The questionnaire was tested before starting the survey, and the questions were adjusted accordingly.

The questions asked aimed at collecting information on farmers, herd demographics, and herd management, besides KAP regarding ticks and tick-borne pathogens. To ensure the anonymity of the interviewed persons according to the national Tunisian regulation [13], all responses were anonymised. All farmers were informed about the aims of the survey, and they gave their verbal consent.

The open-source application Open Data Kit collect (ODK), version 2018 [14] was used to collect answers from animal owners. The questionnaire data were transferred into an Excel sheet, then converted to xml format and uploaded to the ODK application using a tablet. All questions with the corresponding multiple-choice answers are provided in Supplementary Table S1.

### 2.3. Data Analysis

Answers related to knowledge and perception questions were classified as correct or incorrect, according to the state of the art. The answers reflecting attitudes and practices were ranked as positive and negative attitudes. A negative attitude was defined as dangerous and/or incorrect behaviour.

### 2.4. Statistical Analysis

The data were then analysed using SPSS software (version 23, IBM, USA). Averages, frequencies, and associated standard errors and 95% confidence intervals were estimated according to Schwartz [15]. The comparisons between the provided responses or between the groups were tested using a Chi Square or Fisher test for small samples at *p* ≤ 0.05 threshold.

## 3. Results

Eighty-six sheep farmers were included in the present study and answered the questionnaire in an average time of 18.2 ± 14.6 min. The global average response rate to the 40 questions regarding herd management was 91.3 ± 12.2% (Table 1).

### 3.1. Demographic Characteristics of Sheep Farmers

The average age of the questioned farmers was 50.6 ± 15.9 years, with a range of 16–81. The majority of them were men (69.7%; 60/86) and the sex ratio (male/female) was 2.3 (*p* = 0.008). The average number of years of seniority in sheep farming was 23.8 ± 16.5 years (range: 2–60). More than 50% of the respondents received an education and went at least to primary school. The majority (80.3%; 69/86) of the sheep farmers did not practice another activity except farming and 67.5% (58/86) of them inherited breeding activity from their parents, whereas 25.6% (22/86) of the respondents practice sheep farming as the only source of livelihood (Table 2).

### 3.2. Management of Sheep Herds

The main purpose of sheep farming for 81% of respondents was live animal trading, whereas 12.4% were meat producers. Among the 10 farmers who practised transhumance, seven practised it during the summer, two during winter, and one throughout the year. Barbarine and Queue Fine de l’Ouest sheep breeds were kept by 38.1% and 27.7% of the farmers, respectively. The sheep owners had also dogs (28.7%), chickens (27%), and goats (23%).

The family members took care of the animals included the householder (41.5%), his wife (30.7%), and his children (17%), and only 6.8% and 2.3% used employees and shepherds, respectively, to take care of their sheep. The most involved woman in sheep breeding was the farmers’ spouse (70.9%), followed by the mother (32.6%) (Table 3). Among the questioned farmers, 79.1% let their sheep graze on grass (59.8%), spontaneous vegetation (14.7%), or stubble (7.8%). When the sheep were sick, 89.5% of the owners reported calling the veterinarian (Table 3).

The presence of ticks was reported by 87.2% (75/86) and 73.3% (63/86) of the sheep owners on their animals and inside pens or on the ground, respectively. Almost 80.23% (69/86) of the respondents said their animals suffered from tick infestations. According to 68.6% and 23.26% of the questioned sheep owners, summer followed by autumn were the tick seasons (Table 4).

### 3.3. Knowledge, Attitudes, and Practices of Sheep Farmers Regarding Ticks and Tick-Borne Pathogens

**Figure 2:**
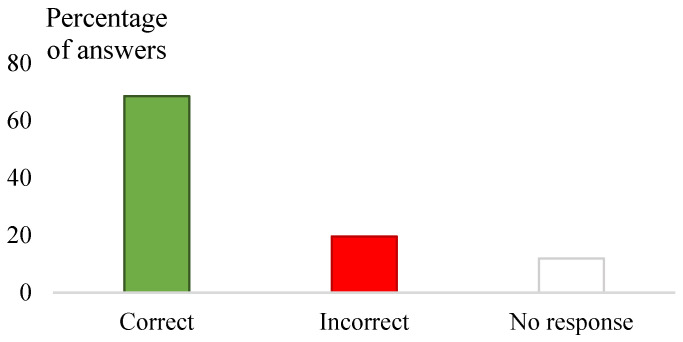
Classification of sheep farmers’ knowledge and perceptions regarding ticks and tick-borne pathogens (the answers are given as percentages among the total answers given for all the knowledge and perception questions). Answers related to knowledge and perception questions were classified as correct or incorrect according to the state of the art.

**Figure 3:**
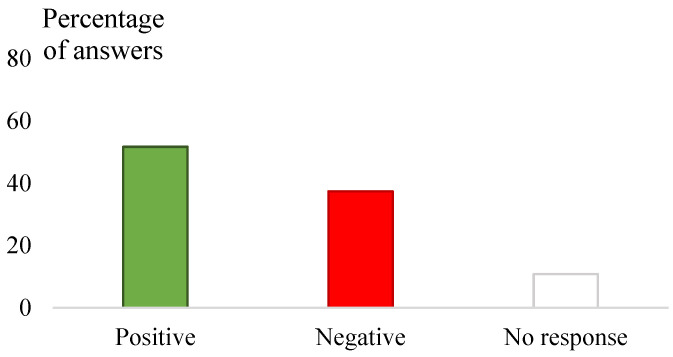
Classification of sheep farmers’ attitudes and practices regarding ticks and tick-borne pathogens (the answers are given as percentages among the total answers given for all the attitudes and practices questions). Negative attitude was defined as dangerous and/or incorrect behaviour, and positive attitude is defined as not harmful and/or protective behaviour.

The sheep owners gave 68.5% (800/1168) of the correct answers (Figure 2) and had 51.7% (643/1242) of positive attitudes regarding the KAP questions, respectively (*p* < 0.0001) (Figure 3). For both types of questions, the percentage of “no response” ranged between 10.9% (135/1242) and 11.9% (139/1168) (Figure 2 and Figure 3).

#### 3.3.1. Knowledge of Sheep Farmers Regarding Ticks and Tick-Borne Pathogens

**Figure 4:**
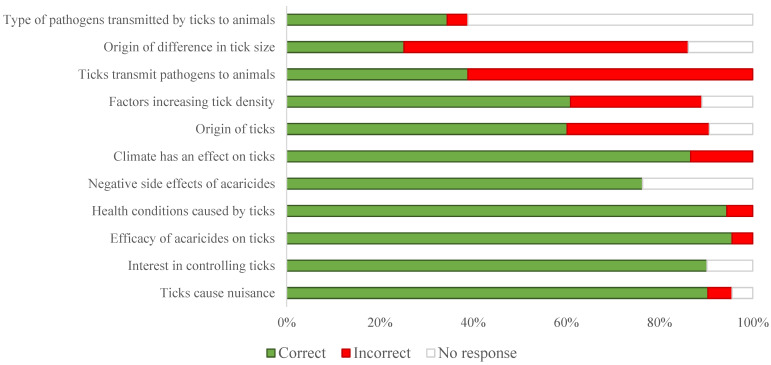
Proportions of correct and incorrect answers to knowledge and perception questions (the answers are given as percentages among the total answers given for each question related to the farmers’ knowledge and perception). All questions with the corresponding answers are provided in Supplementary Table S1.

Almost 82.6% (71/86) of the sheep farmers reported that ticks come from other animal species, whereas 31.4% (27/86) and 24.4% (21/86) thought that ticks come from the soil and grass, respectively. Equally, 16.28% (14/86) reported that ticks come from litter or dirt (Figure 4, Table 5). The majority of the sheep owners (82.6%; 71/86) noted the presence of different tick sizes. Only 29 (33.7%) and 9 (10.5%) out of 86 knew that the difference in size is caused by an engorged state and different tick types, respectively. Almost all the sheep farmers (96.51%; 83/86) knew that ticks cause nuisance for animals, mainly weight loss (58.14%; 50/86), itching, anorexia, oedema (48.83%; 42/86), and skin lesions (23.26%; 20/86). It was evident for 84.88% (73/86) of the sheep owners that controlling ticks improves the health status of their animals. Two main benefits were reported, namely a gain in weight (26.74%; 23/86) and an improvement in animal production (10.47%; 9/86) (Figure 4).

#### 3.3.2. Attitudes and Practices of Sheep Farmers Regarding Ticks and Tick-Borne Pathogen Control

**Figure 5:**
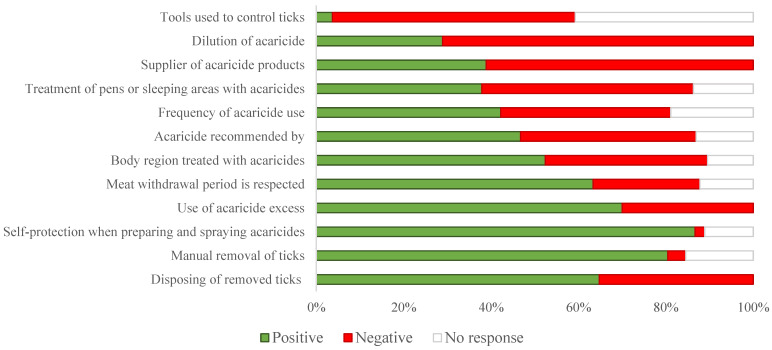
“Positive” and “negative” answers to attitudes and practice questions (the answers are given as percentages among the total answers given for each question related to the farmers’ practices and attitudes). All questions with the corresponding answers are provided in Supplementary Table S1.

Animal treatment (80.2%; 69/86) and pen treatment (61.6%; 53/86) were the main measures adopted by the sheep owners to control ticks. Among the 70.9% (61/86) of respondents that remove ticks manually, 45.3% (39/61) crushed the removed ticks. Out of the 86 interviewed sheep owners, 75 (87.2%) sprayed acaricides on their animals. The acaricide product used was bought mainly (70.9%; 61/86) from a pharmacy. About 9.3% (8/86) and 7% (6/86) of the sheep owners were supplied with acaricides by either regional veterinary services or private veterinarians, respectively (Figure 5, Table 6). The acaricide was recommended by a veterinarian (30.2%; 26/86) or a pharmacist (27.9%; 24/86) but also by friends and neighbours (25.6%; 22/86). Acaricides were used by 40.7% (35/86) of the farmers each time there were ticks on the animals or several times per year (24.4%; 21/86). The majority of the animal owners (72.1%; 62/86) sprayed acaricides on the sheep pens, whereas 27.9% did not (*p* = 0.002). More than half of the sheep owners applied acaricides on the fixation sites (66.3%; 57/86), whereas (33.7%; 29/86) applied it all over the animal’s body (*p* = 0.003).

Only 86% (74/86) of the animal owners diluted the acaricide and less than half of the animal owners (40.7%; 35/86) protected themselves while preparing and spraying acaricides on the animals. The remaining diluted acaricide was either used to treat the pens (40.7%; 35/86), conserved for further use (32.6%; 28/86), or disposed (18.6%; 16/86). Less than half of the respondents (40.7%; 35/86) did not respect the withdrawal period for meat after treating the animals with acaricides (Figure 5, Table 6).

### 3.4. Perception of Sheep Owners Regarding Ticks and Tick-Borne Pathogens

More than half of the sheep owners (61.6%; 53/86) were not aware of the role of ticks as a vector for pathogens. However, 16.3% (14/86) and 17.4% (15/86) of the respondents thought that ticks transmit microbes and cause icterus in sheep, respectively. Among this category of sheep owners (*n* = 29), 21 attended at least to primary school, whereas 8 were illiterate (*p* = 0.05). According to the opinion of 88.4% (76/86) of the sheep owners, the climate has an effect on ticks and heat is the most mentioned (77.9%; 67/86) climatic factor increasing tick density, followed by humidity (16.3%; 14/86) (*p* < 0.001) (Table 6). The majority of the animal owners (97.7%; 84/86) were convinced that acaricides are efficient against ticks and were aware of their negative side effects. Indeed, 61.6% (53/86) and 31.4% (27/86) of the reported negative side effects were toxicity for animals and toxicity for humans, respectively. The role of women in sheep breeding was qualified as very important and important by 84.9 and 14% of farmers, respectively (*p* < 0.001) (Table 6).

## 4. Discussion

A KAP questionnaire was used to assess the knowledge, attitudes, and perception of sheep farmers in Tunisia regarding TBPs. According to Zöldi et al. [16], KAP is an easy tool to assess the population’s awareness regarding ticks and tick-borne diseases. In fact, analysing and understanding the farmers’ behaviours and decision-making drivers is of paramount importance to successfully implementing disease control measures [17].

A high proportion (91.3%) of the sheep farmers surveyed responded to the questionnaire. In Tunisia, most sheep owners are aware of the importance of science in improving animal husbandry and herd management practices. Some of them participate regularly in epidemiological research studies. Sheep owners prefer to talk about their experience and share stories about what they do more than answering questions about KAP. A total of 70% of the sheep farmers were men and only 30% were women. Despite the fact that the surveyed sheep farmer cohort was not randomized, this tendency is concordant with the national data, since women represent only 22.3% of the active workers in agriculture, which demonstrates that farming is still a man’s activity [18]. However, the contribution of women in farming activities is very important, as perceived by the sheep owners in the present study and was mainly the householder’s wife and/or mother. This gender imbalance is prevalent in Tunisia, as women’s efforts in agriculture are still not being recognized. Indeed, according to a survey conducted on 1400 farmers in central Tunisia, women spend more time (4 h) on unpaid work than men, whereas almost the same time (3 h per day) is spent on farming activities by both genders [19]. Tunisia is ranked 5th in the Middle East and North African (MENA) region and 1st in North Africa in gender inequality [20]. More effort is therefore needed to achieve gender equity, since gender inequality does impede human development as stated by the United Nations Development Programme (UNDP) [21].

The high mean age of the sheep farmers, approximately 50 years, could be explained by the disinterest of young Tunisians in all agricultural activities [18]. Most of interviewed sheep farmers reported that farming is the only source of subsistence for them. Indeed, sheep farming in Tunisia is the main source of income for more than 274,000 households [22]. In fact, in 2022, 77.5% of livestock was composed of sheep (4637 thousands of female units), representing approximately 48% of Tunisian red meat production [23].

A high proportion of farmed sheep is destined for live animal sale, mainly in Eid El Idhha (the Muslim sacrifice feast). Indeed, Tunisians prefer sheep meat coming from extensively managed flocks, because the animals are less stressed and graze on natural pastures [24]. Most Tunisian sheep farmers are sedentary and transhumance activities are decreasing generally in the whole country, whereas land surfaces used for large crops are increasing [20,21,25]. The Barbarine sheep breed is the most frequently reared sheep breed among the interviewed sheep owners. This breed is mostly managed under an extensive production system because it is well adapted to the harsh environmental conditions of the country, being tolerant to both hot and cold weather and resistant to low-quality feed and water shortage [26,27]. The presence of other domestic animals such as goats, cattle, and dogs is specific to the mixed sheep–cereal production system [28] maintained by “crop–livestock” farmers [25]. This production system that characterizes mainly Northern and Central Tunisia integrates sheep, goats, and cattle farming with cereal production, mainly wheat and barley. Straw and barley constitute a part of ruminants’ diet [28].

Among the questions about knowledge, 68.3% were answered correctly. This trend is in agreement with that reported by Chakraborty et al. [29], where 60% (30/50) of the respondents had at least moderate knowledge about ticks. More than half of the correct knowledge and positive “attitude” answers could be associated to several factors. On one hand, the duration of experience of the interviewed animal owners, which was on average more than 23 years, could play a role in raising their knowledge. On the other hand, good access to the internet in Tunisia contributes to raising the knowledge and awareness of farmers regarding herd management and the important role of veterinarians. Indeed, in 2022, among 11.9 million Tunisians, more than 8 million (69%) had access to the internet [30]. Moreover, the number and geographic distribution of veterinary surgeons increased during the last three decades in Tunisia, which ensured a better coverage of the rural areas. In fact, from 1994 to 2024, the total number of active veterinary surgeons in Tunisia increased from 466 to 1400, according to the Tunisian National Council of Veterinary Surgeons (personal communication).

According to the present study, a high percentage (89.5%) of the sheep farmers call for veterinary services when their animals are sick. This finding is in contradiction to those of Jeljli et al. [31], who reported a lower percentage of sheep farmers calling for veterinary surgeons in the case of abortion occurrence in Tunisia. This lesser interest in abortion is due to the farmers’ perception that the most frequent causes of abortion are physical factors, such as trauma, climate, and stress, which would not need a veterinary consultation.

Almost all the sheep farmers reported the presence of ticks on and around their animals, confirming that tick infestations are widespread among sheep in Tunisia [11,32,33].

A large proportion of respondents reported that the ticks mainly derive from other animal species. Indeed, seeing ticks on multiple animals and on different animal species, mainly dogs, made farmers think that all these species share the same tick population. To a lesser extent, the farmers reported that ticks come from the soil and grass, probably because they see ticks on the ground. In Bhutan, 41.9% (103/246) of cattle owners declared that the main tick source is the forest [34]. The Tunisian sheep owners did not report the forest as a source of ticks, because its total area is limited to a small region in northern Tunisia.

A large proportion of farmers were aware that ticks affect the animals’ welfare and that they cause also weight loss, itching, skin lesions, anaemia, and general health problems. Weight loss was also reported by 97% and 36% (16/44) of cattle farmers as a consequence of tick infestation in Bhutan [34] and in Algeria [35], respectively. Indeed, the majority of tick-borne pathogens cause weight loss [3,4]. Surprisingly, more than half of the respondents did not know the vectorial role of ticks. This is not in agreement with the findings of Rajput et al. [36] in Pakistan and in Kyeyun, Uganda [37], which reported that 52% and 52.9% of the questioned livestock owners knew about the vectorial role of ticks, respectively. The lack of knowledge of the Tunisian farmers regarding the vectorial role of ticks could be explained by the fact that most of the farmers behave according to their biophysical environment [38] and as microbes are not visible, some farmers are not able to imagine or to figure out the transmission role the ticks have. Moreover, the most prevalent sheep tick-borne agents in Tunisia are *Theileria ovis* and *Babesia ovis* [39], which are not pathogenic, whereas *T. lestoquardi*, the most pathogenic sheep piroplasm, was reported only once by Rjeibi et al. [40].

To control ticks, the sheep owners remove them and spray acaricides on the animals and in their pens. Mechanical tick removal is by far the most used tick control method [41,42,43]. In order to prevent pathogen transmission and secondary infections due to the detachment of the tick rostrum, hand tick removal could be performed with commercial tick removal tools, such fine tipped tweezers or forceps [44], which are not used so far by Tunisian farmers. Awareness and education regarding hand tick removal and the related risks could target all Tunisian animal owners.

A large proportion of the animal owners buy acaricides from their pharmacy with or without a veterinarian prescription. The availability of acaricides with no prescription leads to their misuse, resulting in an increase in genetically resistant tick populations [45]. In Tunisia, acaricides with a veterinary license are available only in pharmacies, while other acaricides are available in phytosanitary shops and are provided by untrained sellers [45]. More than half (59%) of the questioned sheep owners do not protect themselves when diluting the acaricides. The Food and Agriculture Organization developed “Guidelines for personal protection when handling and applying pesticides” [46]. Unfortunately, the farmers have no access to these documents since they are generally in English. Such guidelines should be converted into leaflets and translated into the Tunisian Arabic dialect using iconography.

The toxicity of the acaricides to the animals was two times more frequently reported than their toxicity to humans, which shows clearly that the animal owners are not aware of their own health risk when using acaricides. Indeed, chronic exposure to pesticides impacts the blood cells, the liver, and the peripheral nervous system [47]. Qiao et al. [48] showed that farmers who were exposed to high quantities of pesticides were more likely to report headaches, nausea, and skin problems.

No information was collected on the dilution of the acaricides, and for this reason, we could not conclude whether sheep owners use the correct dilution or not. Moreover, one-third of the questioned sheep owners keep diluted acaricides for future use. Misconceptions regarding acaricide use have been reported in other countries, such as in Ethiopia, where 68.3% (86/120) showed poor acaricide practices [49].

Almost all the farmers (97.7%) think that acaricides are effective against ticks. Similar observations were reported in Finland among the general population living in at-risk areas for tick-borne diseases [16]. This perception is reinforced by the direct action of acaricides on ticks that is visible to farmers immediately when applying the product. Indeed, in a KAP survey, 42.7%, 32.9%, and 23.6% of animal owners reported that ticks detach from the animals after acaricide use within one day, a few hours, and a few days, respectively, according to Namgyal et al. [34,50]. The efficiency of acaricides is also associated with the improvement of the general health status of the treated animals, according to the thoughts of the sheep farmers.

The majority of the sheep farmers think that the climate has an effect on ticks, mainly high temperature. This is true, since Tunisia was classified as a hot spot region for climate change [50]. Among the impacts of climate change, the loss of livestock by a reduction in local food production, an increase in food price, and an increase in pests and diseases are the main consequences [51]. In the last decades, Tunisian agriculture suffered from climate change mainly in the centre and in the south, where droughts occur more frequently than in the North or in the coastal regions [52]. In fact, the increase in tick prevalence and in tick abundance on Tunisian sheep was higher during the summer of 2019 compared to the summer of 2018 [11]. This increase was due to the particular climate of 2019; it was cold and rainy in the winter and spring, with a hot summer ranked as the third hottest one since 1950 [53]. These data show that Tunisia is undergoing a dramatic climatic variation that has a negative impact on livestock farming activities, mainly tick-borne disease occurrence. Global warming is frequently addressed on TV and social media, which may explain the perception of the animal owners towards the negative impact of climate change.

We identified a gap of knowledge and negative attitudes among the sheep owners regarding ticks and tick-borne pathogens. Our findings can be used for awareness communication tools on tick-borne diseases, mainly regarding the vectorial role of ticks and the use of acaricides. In fact, accessing acaricides without a veterinarian prescription, using acaricides repeatedly for treatment, conserving diluted acaricides for deferred use, and preparing acaricides without personal protective equipment are the main gaps. The lack of knowledge about the vectorial role of ticks is also problematic.

Training on ticks and tick-borne infections proved to be effective among extension workers in rural communities in Illinois, USA [54]. According to the authors, the knowledge, attitudes, and practices of the trainees improved dramatically after receiving courses comprising modules on ticks [54]. Similar materials could be produced and adapted for the Tunisian Ministry of Agriculture’s advisors, who are in direct contact with livestock farmers and play a central role in advising farming and agricultural activities.

## 5. Conclusions

As far as we know, this is the first KAP survey about TBPs among Tunisian sheep owners. The findings of the present study could contribute improving our understanding of sheep owners’ KAP regarding TBPs and highlight the necessity and importance of correcting the misconceptions and negative attitudes and practices.

This study shows what farmers actually know about TBPs and how they behave in terms of prevention and treatment. Some knowledge gaps were uncovered, and especially the questions on the use of acaricides and their handling were alarming. The results of this study can be used for communication tools to raise awareness among farmers.

## Figures and Tables

**Table 1:** Average response rate by type of question.

Type of Question (Number of Questions)	Average Response Rate ± SD
Herd management (11)	96.6 ± 6.6
Observation of ticks (5)	93.6 ± 9.91
Knowledge (8)	87.08 ± 5.94
Perception (4)	89.53 ± 22.27
Attitudes and practices (12)	87.94 ± 10.63
Total (40)	91.3 ± 12.24

S.D.: standard deviation.

**Table 2:** Demographic characteristics of sheep farmer participants from Tunisia (*n* = 86).

Demographic Characteristics		Number of Participants (%)	*p*-Value
Gender	Male	60 (69.7)	0.006
	Female	26 (30.3)	
Level of education	No formal education	28 (32.6)	0.01
	Primary school	30 (38.9)	
	High school	19 (22.1)	
	University	1 (1.1)	
	Other	8 (9.3)	
Other activity than sheep breeding	Employee	14 (16.3)	<0.0001
	Trader	3 (3.4)	
	None	69 (80.3)	
Reasons for sheep breeding	Heritage	58 (67.5)	<0.0001
	Source of livelihood	22 (25.6)	
	Absence of other jobs	11 (12.8)	
	Other	1 (1.1)	

**Table 3:** Herd management practices by questioned sheep owners.

Herd Management Practice		Number of Answers (%)	*p*-Value
Purpose for sheep breeding (*n* = 100)	Breeding for live animal trade	85 (81)	<0.001
	Meat production	13 (12.4)	
	Dairy sheep	1 (1)	
	Animal trader	1 (1)	
Herd management (*n* = 86)	Sedentary	75 (87.2)	<0.001
	Transhumance	10 (11.6)	
	Nomadism	0 (0)	
	No response	1 (1.2)	
Season of transhumance or nomadism (*n* = 86)	Not concerned	76 (88.3)	<0.001
	Summer	8 (9.3)	
	Winter	3 (3.5)	
Sheep breeds (*n* = 155)	Barbarine (fat-tail breed)	59 (38.1)	<0.001
	Queue fine de l’ouest (fine-tail breed)	43 (27.7)	
	Cross-breed	32 (20.6)	
	Noire de Thibar (black breed)	19 (12.3)	
	No response	2 (0.8)	
Other animal species (*n* = 244)	Dogs	70 (28.7)	<0.001
	Chickens	66 (27)	
	Goats	56 (23)	
	Cattle	26 (10.7)	
	Equine	21 (8.6)	
	Dromedaries	3 (1.2)	
	No response	2 (0.8)	
Person taking care of animals (*n* = 176)	Householder	73 (41.5)	<0.001
	Wife	54 (30.7)	
	Children	30 (17)	
	Employee	12 (6.8)	
	Associated shepherd	4 (2.3)	
	Other	3 (1.7)	
Grazing (*n* = 86)	Yes	68 (79.1)	<0.001
	No	16 (18.6)	
	No response	2 (2.3)	
Type of grazing (*n* = 102)	Grass	61 (59.8)	<0.001
	Spontaneous vegetation	15 (14.7)	
	Stubble	8 (7.8)	
	No response	18 (17.6)	
Call the veterinarian when your animals are sick (*n* = 86)	Yes	77 (89.5)	<0.001
	No	8 (9.3)	
	No response	1 (1.2)	
Woman playing the most important role in sheep breeding (*n* = 126)	Wife	61 (70.9)	<0.001
	Mother	28 (32.6)	
	Daughter	22 (25.6)	
	Sister	11 (12.8)	
	No response	4 (4.7)	

**Table 4:** Sheep farmers’ observations associated to ticks and tick-borne pathogens.

Question	Answers	Number of Answers (%)	*p*-Value
Presence of ticks on animals (*n* = 86)	Yes	75 (87.2)	<0.0001
	No	11 (12.8)	
Presence of ticks in pens or soil around animals (*n* = 86)	Yes	63 (73.3)	0.001
	No	23 (26.7)	
Health condition caused by ticks (*n* = 86)	Yes	69 (80.23)	<0.001
	No	17 (19.77)	
Season during which the ticks are causing health condition (*n* = 125)	Winter	7 (8.14)	<0.001
	Spring	18 (20.93)	
	Summer	59 (68.6)	
	Autumn	20 (23.26)	
	No response	17 (19.77)	

**Table 5:** Knowledge, attitudes, and practices of sheep owners regarding ticks and tick-borne pathogens in Tunisia.

Question	Answers	Number of Answers (%)	*p*-Value
Knowledge questions			
Origin of ticks (*n* = 86)	Animals (cattle, small ruminants, other)	71 (82.56)	<0.001
	Soil	27 (31.40)	
	Grass	21 (24.40)	
	Dirt	14 (16.28)	
	Litter	14 (16.28)	
	Other	10 (11.63)	
	No response	7 (8.14)	
Origin of difference in tick size (*n* = 86)	Only different in size	63 (73.3)	<0.001
	Engorged/Non-engorged	29 (33.7)	
	Different tick species	9 (10.5)	
	Other	2 (2.3)	
	No response	15 (17.4)	
Ticks cause nuisance (*n* = 86)	Yes	83 (96.51)	<0.001
	No	3 (3.49)	
Health conditions caused by ticks (*n* = 86)	Weight loss	50 (58.14)	<0.001
	Skin lesions	20 (23.26)	
	Anaemia	15 (17.44)	
	Other (itching, oedema, anorexia, mortality)	42 (48.83)	
	No response	6 (6.98)	
Interest of controlling ticks (*n* = 86)	Improve animals’ general status	73 (84.88)	<0.001
	Weight gain	23 (26.74)	
	Improve animal production	9 (10.47)	
	Other	2 (2.33)	
	No response	11 (12.79)	
Attitudes and practices			
Tools used to control ticks (*n* = 86)	Animal treatment	69 (80.2)	<0.001
	Pen treatment	53 (61.6)	
	Manual tick removal	61 (70.9)	
	Other	8 (9.3)	
	Nothing	7 (8.1)	
Manual removal of ticks (*n* = 86)	Yes	61 (70.9)	0.004
	No	25 (29.1)	
Disposing of removed ticks (*n* = 86)	Crushed	39 (45.3)	<0.001
	Other	10 (11.6)	
	Drowned	5 (5.8)	
	Immerged in acaricide	4 (4.7)	
	Burned	3 (3.5)	
	Thrown out	1 (1.2)	
	No response	32 (37.2)	
Supplier of acaricide products (*n* = 86)	Pharmacy	61 (70.9)	<0.001
	Regional veterinary services	8 (9.3)	
	Private veterinarians	6 (7)	
	Weekly market	3 (3.5)	
	Other	12 (14)	
	No response	12 (14)	
Acaricide recommended by	The veterinarian	26 (30.2)	0.01
	The pharmacist	24 (27.9)	
	A neighbour, friend, relative	22 (25.6)	
	Para-veterinarian professional	14 (16.3)	
	Other	4 (4.7)	
	No response	10 (11.6)	
Frequency of acaricide use	When animals are infested by ticks	35 (40.7)	0.01
	Several times a year	21 (24.4)	
	Once a year	10 (11.6)	
	Twice a year	10 (11.6)	
	No response	10 (11.6)	
Treatment of pens or sleeping areas with acaricides	Yes	62 (72.1)	0.002
	No	24 (27.9)	
Body region treated with acaricides	Tick fixation site	57 (66.3)	0.003
	The whole body	29 (33.7)	
	Other	9 (10.5)	
	No response	13 (15.1)	
Dilution of acaricide	Yes	74 (86)	<0.001
	No	1 (1.2)	
	Other	2 (2.3)	
	No response	9 (10.5)	
Self-protection when preparing and spraying acaricides	No	51 (59.3)	0.2
	Yes	35 (40.7)	
Use of acaricide excess	Treat pens	35 (40.7)	<0.001
	Conserved	28 (32.6)	
	Disposed	16 (18.6)	
	Other	2 (2.3)	
	No response	12 (14)	
Meat withdrawal period is respected	No	35 (40.7)	0.08
	Yes	35 (40.7)	
	No response	16 (18.6)	

**Table 6:** Perception of sheep owners regarding ticks and tick-borne pathogens in Tunisia.

Question	Answers	Number of Answers (%)	*p*-Value
Ticks transmit pathogens to animals	No	53 (61.6)	0.13
	Yes	33 (38.4)	
Type of pathogens transmitted by ticks to animals	No response	53 (61.6)	<0.001
	Microbes (bacteria, virus, parasite)	14 (16.3)	
	Icterus	15 (17.4)	
	Other	4 (4.7)	
Climate has an effect on ticks	Yes	76 (88.4)	<0.001
	No	10 (11.6)	
Factors increasing tick density	Heat	67 (77.9)	<0.001
	Humidity	14 (16.3)	
	No response	10 (11.6)	
	Rain	12 (14)	
	Drought	6 (7)	
Efficacy of acaricides on ticks	Yes	84 (97.7)	<0.001
	No	2 (2.3)	
Negative side effects of acaricides	Toxicity to animals	53 (61.6)	<0.001
	Toxicity to humans	27 (31.4)	
	No response	26 (30.2)	
The role of women in sheep farming	Very important	73 (84.9)	<0.001
	Important	12 (14)	
	No response	1 (1.2)	

## Data Availability

Data are available in this manuscript and Supplementary Materials.

## Supplementary Materials

The following supporting information can be downloaded at: https://www.mdpi.com/article/10.3390/vetsci12010002/s1, Table S1: The questionnaire used in the ODK collect application (questions and the multiple-choice answers).
